# Glucose Transporters: Structure, Trafficking, Physiology, and Disease Relevance

**DOI:** 10.3390/cimb48090861

**Published:** 2026-08-25

**Authors:** Cristina Cueto-Ureña, José Manuel Martínez-Martos, María Jesús Ramírez-Expósito

**Affiliations:** Experimental and Clinical Physiopathology Research Group CTS-1039, Department of Health Sciences, School of Health Sciences, University of Jaén, E23071 Jaén, Spain; ccueto@ujaen.es (C.C.-U.); jmmartos@ujaen.es (J.M.M.-M.)

**Keywords:** glucose transporter, SLC2A, SLC5A, GLUT4, SGLT2, insulin signaling, membrane trafficking, glucose homeostasis

## Abstract

Glucose transport across biological membranes in mammals depends primarily on two transporter families: the facilitative glucose transporters of the SLC2 family and the sodium-dependent cotransporters of the SLC5 family. Together, these proteins mediate tissue-specific glucose uptake, epithelial absorption and reabsorption, nutrient sensing, and adaptive responses to metabolic stress. This review summarizes current knowledge of transporter classification, structural mechanism, tissue distribution, intracellular trafficking, and disease associations, with emphasis on GLUT1, GLUT2, GLUT3, GLUT4, SGLT1, and SGLT2. It also discusses less well-characterized members of the extended GLUT family, the therapeutic implications of SGLT inhibition, and unresolved questions regarding transporter specificity. Furthermore, the review integrates emerging evidence on CAR-T cell engineering, Alport syndrome, lupus nephritis, erythropoietic responses, and the reduction in dementia risk associated with transporter modulation.

## 1. Introduction

Glucose is a central metabolic substrate in mammalian physiology, but it does not cross lipid bilayers efficiently by simple diffusion and therefore requires specialized membrane proteins to move across cellular and epithelial barriers [1,2]. In mammals, glucose transport is mediated mainly by two large transporter families: the facilitative glucose transporters encoded by the *SLC2* genes and the sodium-dependent cotransporters encoded by the *SLC5* genes [1,3]. These families differ fundamentally in transport mechanism, energetic coupling, tissue distribution, substrate selectivity, and physiological role [4,5].

The classical view of the field, centered on a few canonical glucose carriers, is now incomplete [6,7]. Several SLC2 family members transport fructose, urate, dehydroascorbate, or myo-inositol rather than glucose alone, and some localize predominantly to intracellular compartments rather than to the plasma membrane [6,7]. Likewise, the SLC5 family includes transporters for vitamins, choline, iodide, monocarboxylates, and myo-inositol, showing that sodium-coupled solute transport extends far beyond intestinal and renal glucose handling [2,3]. At least one member, SGLT3, is better described as a glucose sensor than as a major net glucose transporter [8]. The human genome also encodes a representative of the SWEET family, SLC50A1, which is evolutionarily distinct from both SLC2 and SLC5 and is thought to function primarily in facilitated sugar efflux rather than uptake [3].

Glucose transporters occupy the interface between membrane biophysics and whole-body physiology [9,10]. They influence how nutrients are delivered to the brain, how the liver buffers postprandial and fasting glucose fluxes, how muscle and adipose tissue respond to insulin, and how kidney and intestine accomplish vectorial transepithelial transport [9,10,11]. Their importance influences multiple organ systems and is relevant to the cardiovascular-kidney-metabolic (CKM) syndrome. The structural biology of SGLT2 has advanced [12,13]. Altered transporter function contributes to pathophysiological processes across the CKM syndrome, where reciprocal dysfunction between systemic metabolic flux and organ-specific glucose handling drives disease progression [13,14]. Beyond traditional glycemic control, these proteins are therapeutic targets for interventions that modulate inflammation, oxidative stress, and mitochondrial bioenergetics. This regulation may also involve the gut microbiota and its driven metabolites, reinforcing epithelial barrier integrity and alleviating systemic endotoxemia, thereby impacting various gut-organ axes including the lung, heart, and brain [15]. Consequently, transporter biology is also relevant to neurodegenerative trajectories, oncometabolism, and systemic vascular integrity [16,17,18,19,20].

This review examines glucose transporter families, structural mechanisms, tissue physiology, intracellular sorting, insulin-regulated trafficking, disease relevance, and therapeutic implications. The emphasis is placed on transporters and mechanisms for which the evidence base is strongest, while areas of incomplete or evolving evidence are discussed more cautiously. That balance is particularly important in a field where structural or cell-biological findings can be overextended if they are not interpreted in the context of tissue physiology and human disease.

## 2. Transporter Families

The SLC2 family comprises GLUT1 through GLUT14 as well as HMIT, also referred to as GLUT13 [6,7]. These proteins share a common 12-transmembrane-domain architecture with cytosolic amino and carboxyl termini and belong to the major facilitator superfamily [4,6]. On the basis of sequence relationships, phylogeny, and substrate selectivity, the family is commonly divided into three classes [6]. Class I includes the canonical glucose transporters GLUT1 to GLUT4 together with the testis-associated duplicate GLUT14 [6]. Class II includes proteins with selectivity for fructose, urate, and related hexoses, such as GLUT5, GLUT7, GLUT9, and GLUT11 [7]. Class III includes structurally divergent and often intracellularly localized transporters, including GLUT6, GLUT8, GLUT10, GLUT12, and HMIT/GLUT13 [6,7] (Table 1).

The SLC5 family comprises sodium-coupled solute transporters with a broader substrate range than glucose alone [2,3]. SGLT1 and SGLT2 are the most physiologically important glucose transporters in this family, but other members transport myo-inositol, choline, iodide, vitamins, or monocarboxylates [2,3]. The label “sodium-glucose cotransporter family” can obscure marked functional divergence between individual members. SGLT3 is a particularly important example because it binds glucose but functions primarily as a depolarizing sensor-like protein rather than a high-capacity transporter [8] (Table 2).

**Figure 1:**
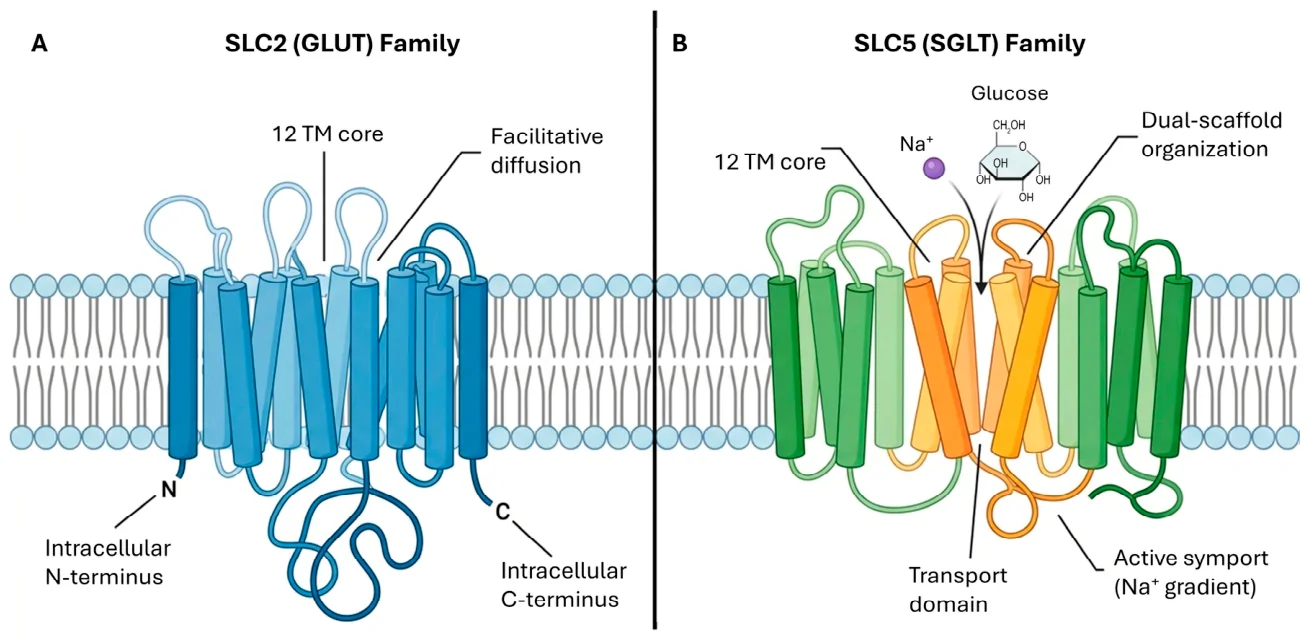
Structural topology and domain organization of mammalian facilitative and active glucose transporters. (**A**) The facilitative SLC2 (GLUT) family. (**B**) The sodium-dependent SLC5 (SGLT) family. Both families have a core architecture of 12-transmembrane domains. In GLUTs, the amino (N) and carboxyl (C) termini face the cytoplasm. SGLT members have a dual-scaffold and transport domain organization for active cotransport against an electrochemical gradient. Abbreviations: C, carboxyl terminus; GLUT, glucose transporter; N, amino terminus; SGLT, sodium-glucose cotransporter; TM, transmembrane domain.

SLC2 and SLC5 transporters are functionally complementary in epithelial tissues [2,10]. In the intestine and kidney, sodium-coupled apical uptake is linked to basolateral efflux through facilitative transporters, classically GLUT2, thereby enabling vectorial transepithelial glucose movement [5,10] (Figure 1).

### Species Differences in Pancreatic β-Cells

Transport physiology studies have revealed interspecies divergence between rodent models and human physiology, particularly within pancreatic β-cells [21,22]. In rodents, pancreatic β-cells express high levels of GLUT2, a relatively low-affinity, high-capacity transporter that allows rapid equilibration of extracellular and intracellular glucose [11,21,22]. In human β-cells, by contrast, GLUT2 expression is much less prominent, often subdominant or difficult to detect, whereas GLUT1 and, to a lesser extent, GLUT3 appear to mediate most glucose entry [21,22].

This divergence has mechanistic and experimental implications. Human β-cells exhibit a lower apparent Michaelis constant and lower maximal transport rate than rodent β-cells, reflecting a system tuned differently for glucose uptake [21,22]. Human β-cells are also relatively resistant to alloxan, the classic rodent β-cell toxin that enters through GLUT2, illustrating how transporter expression can shape experimental vulnerability [21,22]. Yet despite these differences, both rodent and human β-cells rely primarily on glucokinase as the dominant rate-limiting glucose sensor for insulin secretion, because transport capacity still exceeds phosphorylation capacity under most conditions [22]. Accordingly, rodent GLUT2 biology remains informative, but it should not be used as a direct surrogate for human β-cell transporter composition. Recent evidence indicates that β-cell functional integrity and glucose-stimulated insulin secretion (GSIS) are also contingent upon the mechanical and structural scaffold of the cell. Specifically, keratin 8 (K8) has been identified as a critical determinant for the proper plasma membrane targeting of GLUT2; its loss leads to fragile islets, disjointed membrane organization, and impaired GSIS due to transporter mistargeting [23]. These findings indicate that transporter efficiency in pancreatic islets is not solely a function of expression levels but is strictly regulated by the epithelial intermediate filament network [23].

**Table 1:** Classification, substrate specificity, tissue distribution, and pathophysiological relevance of the mammalian SLC2 (GLUT) facilitative hexose transporter family.

Isoform	Gene Symbol	Functional Class	Key Substrates	Main Expression Sites	Physiological/Pathological Relevance
GLUT1	SLC2A1	Class I [6]	Glucose, galactose, dehydroascorbate [7]	Erythrocytes, blood–brain barrier, microvascular endothelium [24]	Basal glucose uptake; pathogenic variants cause GLUT1 deficiency syndrome [4,24,25]
GLUT2	SLC2A2	Class I [6]	Glucose, fructose, galactose, glucosamine [11]	Liver, intestine, kidney, rodent pancreatic β-cells [11,21]	Bidirectional hepatic flux, epithelial basolateral exit, glucose sensing; variants cause Fanconi-Bickel syndrome [2,11,26]
GLUT3	SLC2A3	Class I [6]	Glucose, galactose [7]	Neurons, brain parenchyma [27]	Supports high neuronal glucose demand; disease relevance varies by context [27]
GLUT4	SLC2A4	Class I [6]	Glucose [28]	Skeletal muscle, cardiac muscle, adipocytes [28]	Principal insulin-responsive transporter; defective trafficking contributes to insulin resistance [9,29]
GLUT5	SLC2A5	Class II [6]	Fructose [10]	Small intestine, testes, adipose tissue, skeletal muscle [7]	Major facilitative fructose transporter [10,30]
GLUT6	SLC2A6	Class III [6]	Likely intracellular hexose transport [7]	Lysosome-related compartments, inflammatory macrophage systems [7]	Still incompletely defined physiologically [7]
GLUT7	SLC2A7	Class II [6]	Glucose, fructose [7]	Small intestine, testes, colon, prostate [7]	Physiological role remains incompletely resolved [7]
GLUT8	SLC2A8	Class III [6]	Intracellular hexose-related transport [7]	Endosomes, trans-Golgi network, testes [7]	Strong intracellular targeting; plasma membrane role remains limited [7]
GLUT9	SLC2A9	Class II [6]	Urate, fructose, glucose [31]	Kidney, liver, placenta [31]	Major urate transporter; variants linked to hypouricemia and gout-associated phenotypes [31,32]
GLUT10	SLC2A10	Class III [6]	Glucose, dehydroascorbate [7]	Connective tissues, blood vessels [7]	Associated with arterial tortuosity syndrome; may participate in dehydroascorbate transport and redox-related intracellular processes [33,34].
GLUT11	SLC2A11	Class II [6]	Glucose, fructose [7]	Heart, skeletal muscle [7]	Tissue-specific role remains underdefined [7]
GLUT12	SLC2A12	Class III [6]	Glucose [7]	Heart, prostate, skeletal muscle [7]	Investigated in metabolism and cancer, but evidence remains limited [16,35,36]
HMIT/GLUT13	SLC2A13	Class III [6]	Proton/myo-inositol [6]	Brain, neuronal membranes [6]	Better classified as a proton-coupled myo-inositol transporter [6]
GLUT14	SLC2A14	Class I [6]	Glucose [7]	Testes [7]	Testicular duplicate related to GLUT3 [7]

## 3. Structural Mechanisms of Transport

### 3.1. Alternating-Access Model

Structural work across both facilitative and sodium-coupled transporters strongly supports an alternating-access mechanism [1,4]. In this framework, the substrate-binding site alternates between outward-facing, occluded, and inward-facing states and is not accessible to both sides of the membrane simultaneously. [4]. This principle prevents formation of a permanent aqueous pore and allows selective translocation to occur through sequential conformational changes [1].

For SLC2 transporters such as GLUT1, GLUT3, and GLUT5, available crystal and cryo-EM structures have captured several of these conformational states directly, providing a structural basis for long-standing transport models [4,37]. In SLC5 symporters, the transport cycle is driven by an elevator-type mechanism, where the substrate-binding mobile transport domain (TD) undergoes a large-scale rigid-body translocation relative to a static scaffold domain [38,39]. Recent cryo-EM evidence has identified a novel intermediate conformational state, often terminally ‘stalled’ by bulky ligands or specific lipid interactions in the glucose-binding site, which provides a structural blueprint for the rational design of next-generation, high-affinity transport inhibitors [39]. Although the general framework is robust, important uncertainties remain regarding the kinetics of state transitions, the influence of local membrane composition, and the extent to which particular intermediate states are stabilized or disfavored by specific ligands or partner proteins [1].

### 3.2. Rocker-Switch Versus Elevator Transport

Facilitative GLUT transporters are commonly described by a rocker-switch mechanism [4]. In this model, the N-terminal and C-terminal six-helix bundles pivot relative to one another around a central substrate-binding cavity [1,4]. Substrate access from one side of the membrane is coupled to closure on the opposite side, yielding outward-open, occluded, and inward-open states in sequence [4]. This representation is especially useful for class I GLUTs and remains one of the clearest mechanistic templates in the SLC2 field [1,4].

**Figure 2:**
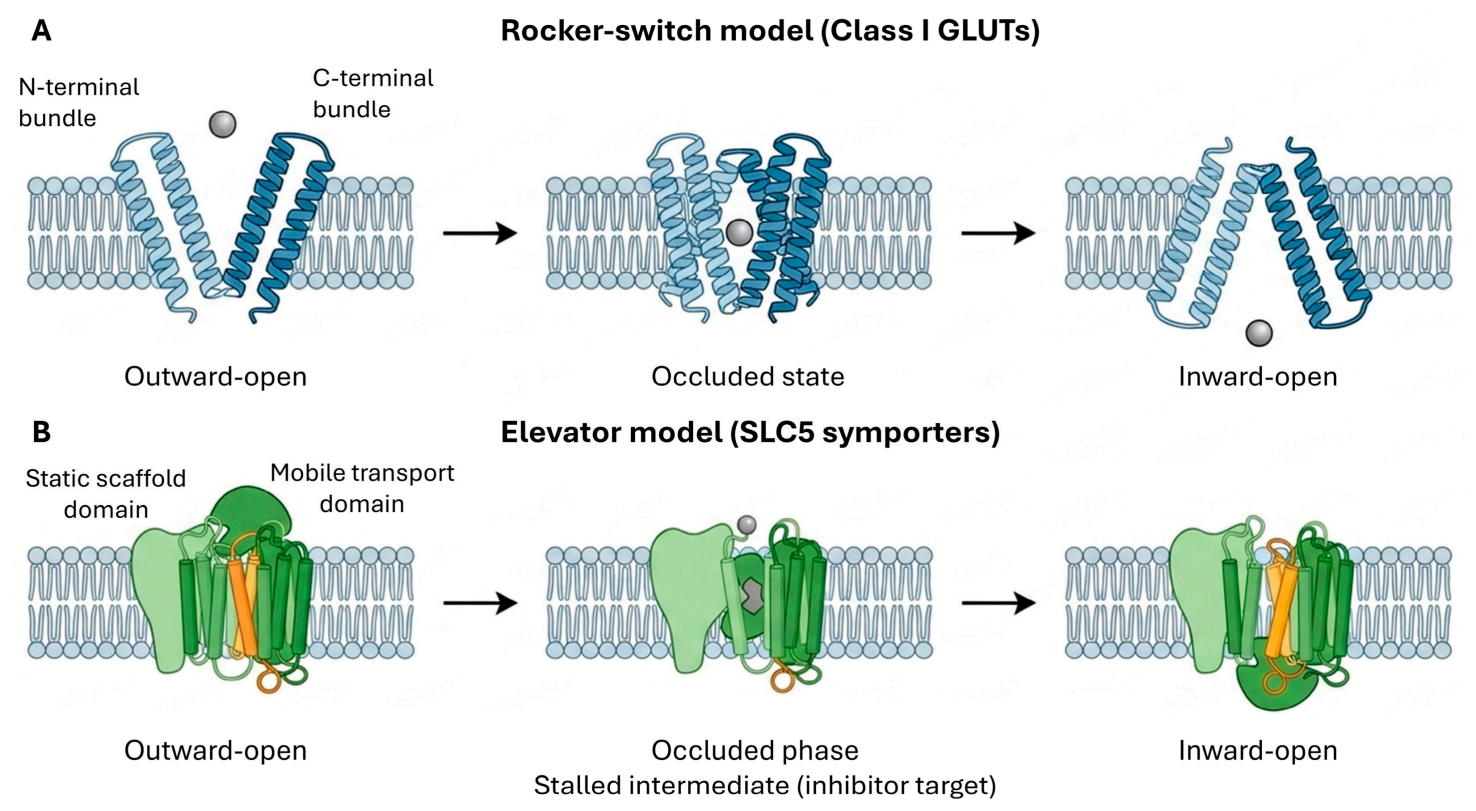
Mechanistic models of substrate translocation across membranes. (**A**) The Rocker-Switch model, characteristic of Class I GLUTs. N-terminal and C-terminal six-helix bundles pivot around a central substrate-binding cavity, cycling through outward-open, occluded, and inward-open states. (**B**) The Elevator model, typical of many SLC5 symporters. A mobile transport domain moves relative to a static scaffold domain. Stalled intermediate states in the occluded phase are possible targets for high-affinity inhibitors. Abbreviations: GLUTs, glucose transporters.

By contrast, several sodium-coupled cotransporters and bacterial homologs are better represented by an elevator-type mechanism [1]. Here, the transporter contains a relatively static scaffold domain and a mobile transport domain that undergoes a larger translational movement within the membrane [1]. The transport domain carries the substrate-binding site and slides relative to the scaffold to expose the bound substrate alternately to either side of the membrane [1]. Although this distinction can sound schematic, it has significant implications for understanding inhibitor binding, ion coupling, conformational energetics, and the structural logic of domain organization [1]. A bacterial model helps clarify this point. Structural work on bacterial systems such as glucose-specific permease components within phosphoenolpyruvate-dependent phosphotransferase pathways has illustrated how domain mobility can be captured experimentally in defined conformational intermediates [5]. In such systems, substrate translocation may be coupled directly to chemical modification, unlike mammalian GLUTs or SGLTs, yet the broader structural lesson remains valuable: transport is often best understood as orchestrated domain movement rather than a simple opening and closing of a static pore [5] (Figure 2).

### 3.3. Gating Networks and Oligomeric Dynamics

The alternating-access cycle of facilitative transporters depends not only on large domain movements but also on local gating interactions [4,40,41]. In GLUT1, molecular dynamics and structural analyses indicate that extracellular gating involves conserved residues distributed across transmembrane segments including TM1, TM5, TM7, and TM8, which participate in rearrangements that reduce energetic barriers to substrate entry [41]. Intracellular opening is controlled by additional salt-bridge and loop interactions, especially involving the long intracellular loops and the cytoplasmic faces of the transmembrane helices [41].

These local interactions matter because they provide a mechanistic explanation for how closely related transporters can display distinct transport rates, inhibitor sensitivities, or substrate preferences despite sharing a broadly similar fold [4,41]. Gatekeeper residues can influence not only the existence of open and closed states but also the relative dwell time in each state. In some transporters, aromatic side chains and charged residues near the internal vestibule appear particularly important for coordinating opening on the cytoplasmic side [41].

GLUT1 has also been described as a dynamic noncovalent oligomer, forming dimers and tetramers rather than functioning exclusively as an isolated monomer. Some biochemical studies have suggested trans-allosteric behavior within the tetrameric state, in which occupancy of one subunit can influence the conformational state or affinity of neighboring subunits [42,43]. Inhibitor studies using endofacial ligands such as forskolin or cytochalasin B have been interpreted in this framework. These findings remain mechanistically interesting, but their physiological significance in intact tissues is not fully settled and should not be generalized too broadly beyond the systems in which they were measured [42,43].

### 3.4. Cryo-EM Structures in the SLC2 Family

Single-particle cryo-EM has expanded structural resolution across the SLC2 family, especially for transporters that had been difficult to characterize by crystallography alone [40,41]. Human GLUT4 has been resolved in an inward-open conformation bound to cytochalasin B, revealing a transmembrane architecture highly similar to GLUT1 but with meaningful isoform-specific differences. The inhibitor is coordinated through a combination of polar and hydrophobic contacts, and subtle residue substitutions help explain why related transporters can differ in inhibitor orientation or binding geometry even when their overall fold is strongly conserved [41].

Cryo-EM studies have also informed the interpretation of GLUT7 and GLUT9. GLUT7 has been captured in an outward-open apo conformation in lipid nanodiscs, consistent with a canonical MFS fold but with features compatible with transport of both glucose and fructose [44]. GLUT9, in contrast, has been resolved with urate or the competitive inhibitor apigenin, revealing a more polar substrate-binding cleft than classical glucose-preferring transporters and supporting its established role in urate handling [45]. These structures show that the SLC2 family should not be conceptualized merely as a series of near-duplicate glucose channels. Instead, distinct members have adapted the same general architectural framework to support substantially different physiological tasks [44,45].

### 3.5. SGLT2-MAP17 Assembly and Gating

Cryo-EM studies have resolved the structure of the human SGLT2-MAP17 complex [38]. Rather than functioning as a completely autonomous monomer, SGLT2 forms a heteromeric complex with the single-pass membrane protein MAP17, which contributes to structural stabilization [38]. In current models, the MAP17-associated bundle contributes to a scaffold-like framework, whereas the mobile core elements surrounding the sodium- and glucose-binding sites undergo conformational transitions during the transport cycle [38].

Sodium binding stabilizes the outward-open state and enhances substrate affinity, while subsequent substrate binding and domain rearrangement permit inward transition and solute release [38]. This framework links transporter physiology directly with inhibitor pharmacology [38]. The clinically successful gliflozin class can be understood structurally as taking advantage of conformational states and access pathways specific to SGLT2, helping to explain high potency and relative selectivity [38].

SGLT1, although structurally related, is not merely a less renal version of SGLT2. The cryo-EM structure of human SGLT1 has elucidated the molecular basis for its high sugar affinity and water permeability, confirming a conserved core architecture shared with SGLT2 but with distinct extracellular loops and substrate-binding pocket refinements [2,3,46]. It has distinct kinetic properties, broader physiological distribution, and additional channel-like behavior that has been proposed to support passive water and urea movement across the enterocyte apical membrane [3]. This property has been proposed in the context of oral rehydration therapy, where coupled sodium-glucose uptake and associated water movement are central to intestinal fluid absorption [3]. Even so, its primary importance remains as a sodium-coupled transporter of glucose and galactose [3,10].

### 3.6. SGLT3 Sensor Mechanics

SGLT3 is one of the clearest examples of functional divergence within the SLC5 family [8]. Although it shares substantial sequence similarity with SGLT1, human SGLT3 does not appear to perform substantial net sugar transport [8]. Instead, glucose binding induces sodium-dependent membrane depolarization, making the protein better described as a sensor or sensor-like transporter [8].

Mechanistically, this divergence has been linked to specific substitutions in residues involved in sugar coordination and coupling [8]. Work centered on transmembrane segment 11 has suggested that alteration of a conserved glutamine found in SGLT1 and SGLT2 can decouple substrate binding from productive translocation [8]. The resulting phenotype is especially informative because it shows that within a conserved structural scaffold, small sequence changes can shift function from transport to sensing without abolishing ligand recognition altogether [8].

### 3.7. G6PT Transporter (SLC37A4)

Cryo-EM studies resolved the human glucose-6-phosphate transporter (G6PT/SLC37A4), which functions as a sugar-phosphate/inorganic phosphate (Pi) antiporter [47,48]. The structures in apo, Pi-bound, and substrate-bound states reveal that Pi binding triggers an interdomain salt bridge formation that compacts the central cavity for G6P recognition, orchestrating its translocation into the endoplasmic reticulum lumen—a mechanism essential for systemic glucose homeostasis and whose dysfunction drives glycogen storage disease type Ib [47,48]. Beyond metabolic homeostasis, G6PT has been identified as a critical host factor in viral pathogenesis. For instance, porcine reproductive and respiratory syndrome virus (PRRSV) upregulates G6PT expression to facilitate replication by suppressing RIG-I- and TLR3-mediated type I interferon transcription, while simultaneously enhancing aerobic glycolysis to meet the energetic demands of viral propagation [49]. These findings identify G6PT as a potential target for both metabolic and anti-viral therapeutic strategies.

## 4. GLUT1 and GLUT3 in the Brain

GLUT1 and GLUT3 form a functional sequence for glucose delivery from blood to neurons [25,50,51,52]. GLUT1 is highly expressed in endothelial cells of the blood–brain barrier and contributes substantially to glucose entry into brain parenchyma, whereas GLUT3 is enriched in neurons and has kinetic properties suited to high neuronal glucose demand [25,50,51,52]. This arrangement remains one of the most important examples of tissue specialization in the SLC2 family [27].

Beyond constitutive glucose delivery, the field has transitioned toward a model of dynamic, activity-dependent regulation through the recently characterized neuroimmune metabolic circuit [53]. In this paradigm, microglia function as essential metabolic sensors that monitor neuronal activity; upon increased demand during cognitive or motor tasks, microglia secrete the hypoxia-responsive protein CYR61, which actively upregulates glucose transporter expression in the brain vasculature [18,53,54]. This microglial-vascular coupling ensures that substrate delivery is precisely calibrated to support on-demand protein synthesis and synaptic plasticity, moving the narrative beyond a simple blood-to-neuron gradient [53].

**Figure 3:**
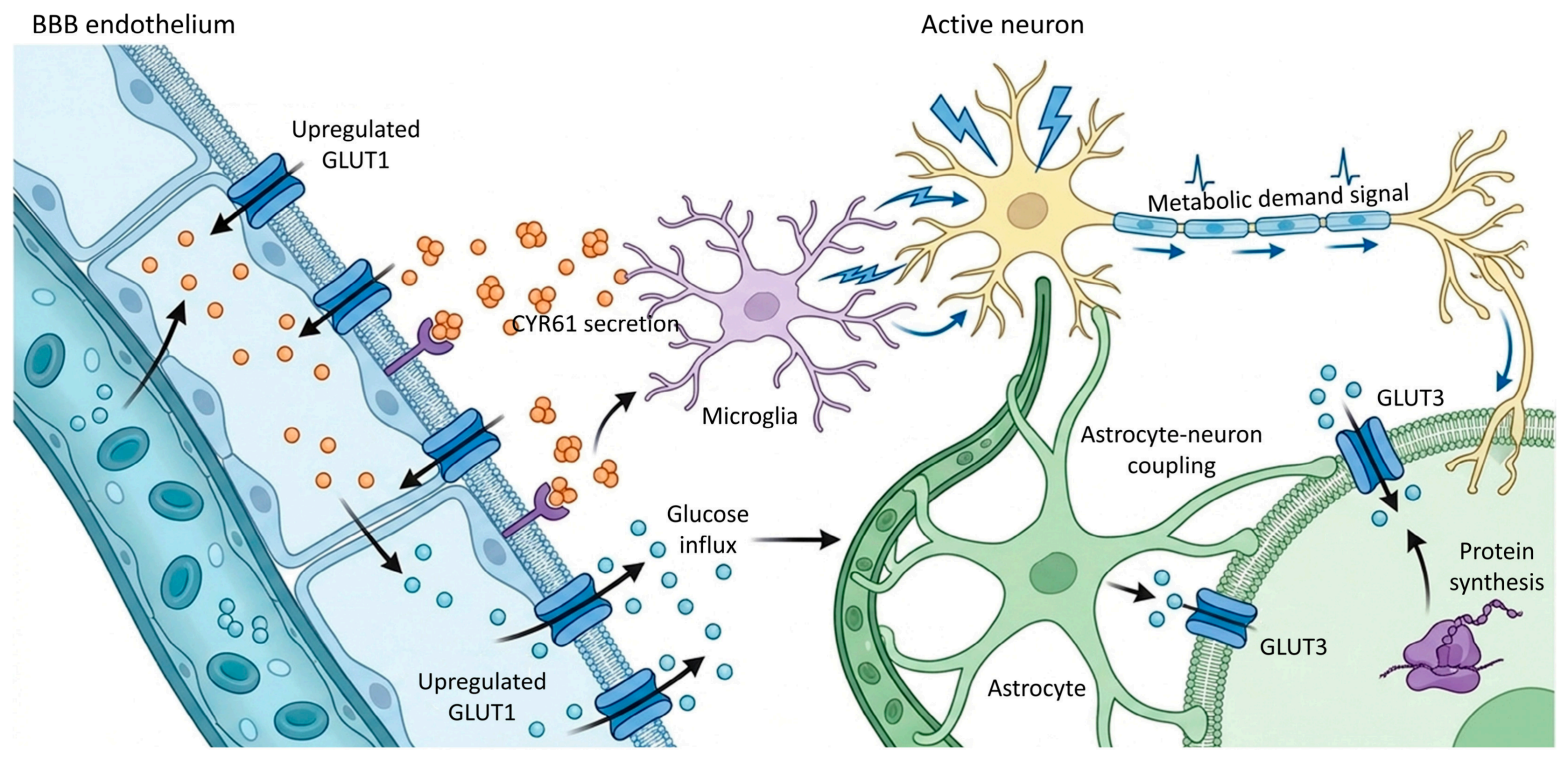
Activity-dependent regulation of cerebral glucose delivery via the neuroimmune metabolic circuit. Neuronal activation triggers microglial sensing of metabolic demand. Microglia secrete the hypoxia-responsive protein CYR61. CYR61 upregulates GLUT1 expression in brain endothelial cells of the blood–brain barrier (BBB), increasing glucose supply for neuronal demand and protein synthesis. Astrocyte-neuron metabolic coupling and the constitutive role of neuronal GLUT3 are represented. Abbreviations: BBB, blood–brain barrier; CYR61, cysteine-rich angiogenic inducer 61; GLUT, glucose transporter.

Developmental work suggests that GLUT1 expression correlates more strongly with general nutrient supply and tissue growth, while GLUT3 tracks neuronal maturation and activity more closely [27]. These observations indicate that transporter expression can reflect not only local substrate availability but also developmental programs and cell-type-specific metabolic requirements [27]. Regional brain metabolism and substrate flexibility are increasingly recognized as products of a sophisticated neuroimmune metabolic circuit. For instance, microglial activation during increased metabolic demand triggers the secretion of CYR61, a hypoxia-responsive protein that actively upregulates glucose transporter expression in the brain vasculature to fuel on-demand protein synthesis in active neurons [53]. Furthermore, GLUT1-dependent mechanisms are essential for maintaining endothelial homeostasis under hyperglycemic stress; however, this protective response is compromised in chronic diabetes, where GLUT1 and TXNIP enrichment drives NLRP3 inflammasome activation and vascular permeability [27,55] (Figure 3).

The clearest disease association is GLUT1 deficiency syndrome, caused by pathogenic variants in SLC2A1 [50,52]. Hallmark findings include low cerebrospinal fluid glucose and a spectrum of neurological manifestations that may include seizures, developmental delay, movement abnormalities, and cognitive impairment [24]. A caveat is that some pathogenic variants do not abolish all peripheral measures of transporter function [25]. Relatively preserved erythrocyte transport does not necessarily exclude major physiological impairment at the blood–brain barrier, so peripheral assays must be interpreted in context [25].

The role of GLUT3 in neurological disease is less definitive [27]. Altered expression has been described in several pathological contexts, including injury and neurodegenerative states, but the distinction between causal contribution, compensatory adaptation, and epiphenomenon varies by model [27]. GLUT3 should therefore be treated as biologically important in neuronal metabolism, but not as a uniformly validated disease driver across all neurological disorders [27].

## 5. GLUT2 in Liver, Intestine, Kidney, Pancreas and the Nervous System

GLUT2 occupies a distinctive place among mammalian facilitative transporters because it combines relatively low apparent affinity with high transport capacity and broad tissue distribution [11]. It is expressed in liver, intestine, kidney, pancreatic islets in some species, and selected glucose-sensing cells in the nervous system, participating in both metabolic flux and nutrient sensing [11].

In the liver, GLUT2 supports bidirectional glucose movement under fed and fasting conditions [11]. During the absorptive state, it facilitates hepatic glucose uptake; during fasting, it permits hepatic glucose release generated by glycogenolysis and gluconeogenesis [11]. Its low-affinity, high-capacity behavior is well suited to a tissue that buffers large changes in portal glucose concentration [11]. In pancreatic and extra-pancreatic sensing networks, GLUT2 participates alongside glucokinase and downstream signaling pathways in glucose detection, though its exact physiological weighting differs markedly by species and cell type [11,22].

In the intestine and kidney, GLUT2 is well established as a major basolateral exit pathway [2,10]. Enterocytes rely on it to transfer glucose absorbed apically into the portal circulation, and proximal tubular epithelial cells use it to return reabsorbed glucose to blood [2,10]. By contrast, the concept of major acute apical recruitment of intestinal GLUT2 under high luminal glucose conditions remains more debated [10]. Earlier models proposed that very high luminal sugar loads could drive substantial translocation of GLUT2 to the brush border, augmenting absorptive capacity beyond that provided by SGLT1 alone [10]. Subsequent experimental work has supported a dominant role for SGLT1 even under high-glucose conditions, and the magnitude and generality of acute apical GLUT2 recruitment remain uncertain [5,10]. Current evidence supports presenting this issue as historically important but unresolved rather than as a settled fact [10].

GLUT2-dependent sensing in the nervous system has been linked to feeding behavior, autonomic responses, glucagon secretion, and related homeostatic circuits [11]. These associations are biologically plausible and supported in several experimental systems, but translation to human pathophysiology remains incomplete [11]. As with pancreatic β-cells, species divergence and regional cellular heterogeneity complicate straightforward interpretation [11,22].

Pathogenic variants in SLC2A2 cause Fanconi-Bickel syndrome [2,26]. The disorder combines hepatomegaly, disturbances in glucose and galactose handling, glucosuria, and proximal tubulopathy, reflecting the broad physiological footprint of GLUT2 across epithelial and metabolic tissues [2,26]. The clinical phenotype illustrates an important principle: when a transporter sits at a convergence point between organ systems, the resulting disease may not resemble an isolated tissue-specific defect [26].

## 6. GLUT4 and Insulin-Regulated Trafficking

GLUT4 is the principal insulin-responsive glucose transporter in adipocytes and skeletal muscle and a major determinant of postprandial glucose disposal [9,28]. Its biology differs from that of many other transporters because regulation depends less on total protein abundance than on rapid and highly organized intracellular trafficking [9]. Under basal conditions, most GLUT4 resides in intracellular compartments; insulin sharply increases surface exposure by mobilizing transporter-containing vesicles toward and through the plasma membrane [9,29].

This system is an example of regulated membrane traffic, integrating receptor signaling, vesicle sorting, tethering, cytoskeletal transport, Rab GTPase circuits, and SNARE-mediated fusion [9,29]. It also carries major pathophysiological importance, since insulin resistance can arise from defects at multiple levels of this pathway even when total GLUT4 protein is not profoundly reduced [9].

### 6.1. GLUT4 Storage Vesicles and Sorting

A substantial fraction of intracellular GLUT4 is concentrated in specialized GLUT4 storage vesicles, usually referred to as GSVs [9,56,57]. These vesicles are functionally distinct from the general recycling endosome system because they support a large, rapid, insulin-sensitive increase in cell-surface GLUT4 [9,56]. The GSV concept is strongly supported, but its exact compartmental boundaries, composition, and biogenetic pathways are still being refined [56,57]. Different imaging platforms, cell types, and fractionation strategies do not always isolate the same vesicle populations with equal specificity [57].

Under basal conditions, GLUT4 is actively sorted away from generic recycling routes into small specialized vesicles and tubules, thereby maintaining low plasma membrane exposure [9]. Two cytoplasmic sorting motifs within GLUT4 are especially important in this process: the N-terminal FQQI motif and the C-terminal TELE motif [9,58]. These signals influence retrieval, endosomal sorting, and entry into insulin-responsive compartments rather than merely controlling steady-state localization in a passive manner [58].

The insulin-regulated aminopeptidase IRAP is a key companion of GLUT4 within this network [58]. IRAP colocalizes and traffics with GLUT4, and its depletion disrupts sorting from endosomal pathways into specialized insulin-responsive compartments, thereby increasing basal cell-surface GLUT4 [58]. This result is mechanistically important because it indicates that some GSV components are not just passive cargo markers but active determinants of compartment identity and flux [58].

Recent work has also supported a more distributed trafficking architecture in which specialized storage compartments interact dynamically with endosomal intermediates [57]. This view does not invalidate the classical GSV framework; rather, it suggests that GSVs should be understood as part of a connected network rather than as completely isolated vesicles with a single biogenetic origin [57]. A synthesis of current evidence suggests that insulin-responsive GLUT4 trafficking includes both highly specialized storage elements and broader endosomal routes whose relative contribution depends on cell type and experimental context [9,57].

### 6.2. The TUG Anchoring Cascade

A major mechanism for retaining GLUT4-containing vesicles in the basal state involves the anchoring protein TUG [9,59]. TUG helps tether a subset of GSVs near the trans-Golgi network and the ER-Golgi intermediate compartment, thereby limiting their constitutive movement to the cell surface [59]. In this way, the cell maintains a large intracellular reserve of transport-competent GLUT4 that can be mobilized rapidly upon insulin stimulation [9].

The TUG pathway has been worked out in considerable mechanistic detail [59]. Basally, the Golgi-associated protein PIST binds TUG and suppresses its cleavage [59]. Insulin signaling activates the small GTPase TC10α, which in turn interacts with PIST and relieves inhibition of the protease USP25m [59]. USP25m cleaves TUG at a defined peptide bond, generating a C-terminal Golgi-associated fragment and an N-terminal product, often termed TUGUL, that remains associated with the vesicle [59]. The vesicle-bound fragment has been proposed to modify or recruit the kinesin motor KIF5B, thereby promoting microtubule-based transport of GLUT4 vesicles toward the cell periphery [59].

**Figure 4:**
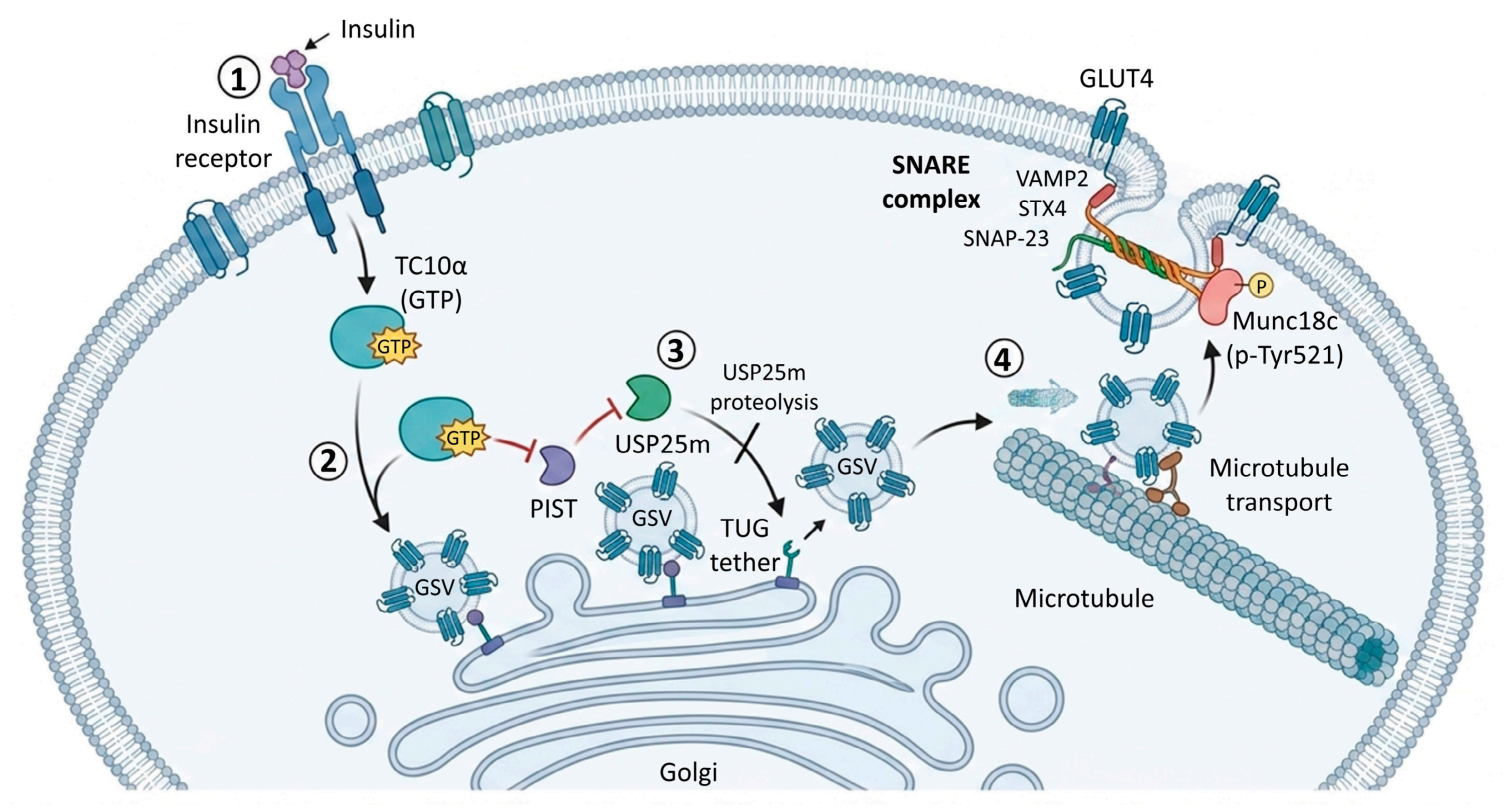
Molecular pathways governing insulin-stimulated GLUT4 translocation. In the basal state, GLUT4 is sequestered in specialized GLUT4 storage vesicles (GSVs) anchored to the Golgi matrix by TUG. Insulin signaling (1) activates the small GTPase TC10α, which (2) relieves PIST-mediated inhibition of the protease USP25m (3). Proteolysis of TUG releases GSVs for microtubule-based transport and subsequent SNARE-mediated fusion at the plasma membrane, a process regulated by Munc18c phosphorylation at Tyrosine 521 (4). Abbreviations: GLUT4, glucose transporter 4; GSV, GLUT4 storage vesicle; PIST, Golgi-associated PDZ-interacting protein; SNARE, soluble N-ethylmaleimide-sensitive factor attachment protein receptor; TUG, tether containing UBX domain for GLUT4; USP25m, ubiquitin-specific protease 25m.

This cascade links receptor-proximal signaling to the physical release and directed movement of specialized vesicles [9,59]. Even so, some features have been defined most clearly in selected cellular models, and the degree to which every component contributes equivalently in adipocytes and muscle cells remains an active area of investigation [59]. The safest formulation is that TUG-mediated anchoring and regulated proteolysis represent an important mechanism for basal retention and insulin-triggered mobilization of a subset of GLUT4 vesicles [9] (Figure 4).

### 6.3. Rab GTPase Sorting Networks

Rab GTPases provide a second major regulatory layer in GLUT4 traffic [9]. In the basal state, AS160/TBC1D4 acts as a Rab GTPase-activating protein that maintains downstream Rabs in an inactive GDP-bound state [9]. Insulin activates Akt, which phosphorylates AS160 and attenuates its GAP activity, thereby allowing target Rabs to accumulate in an active GTP-bound form and promote vesicle mobilization and fusion [9]. This is a well-established link between insulin signaling and membrane traffic [9].

Comparative studies have suggested that different Rab proteins regulate distinct GLUT4 subroutes [9,60]. Rab10 is enriched in transferrin receptor-negative, more specialized GLUT4 storage vesicles and appears especially important for direct insulin-responsive exocytosis [60]. Rab14, by contrast, localizes more strongly to transferrin-positive endosomal compartments and may regulate trafficking through the broader recycling system [60]. Experimental knockdown of both Rab10 and Rab14 produces additive defects, supporting the idea that direct exocytosis from specialized GSVs and recycling through endosomal routes contribute in parallel to the final increase in surface GLUT4 [60].

This distinction explains why GLUT4 translocation should not be described as a single linear pathway [9,60]. Instead, insulin-responsive surface delivery appears to arise from coordinated contributions of multiple intracellular compartments whose routing and fusion efficiencies differ [9,57]. Such a model also helps explain why partial defects in one branch can be partly compensated, whereas combined perturbations produce more severe impairment [60].

### 6.4. SNARE Assembly and Munc18c Gating

The final docking and fusion of GLUT4 storage vesicles with the plasma membrane require formation of a trans-SNARE complex composed of the vesicle v-SNARE VAMP2 and the plasma membrane t-SNAREs syntaxin-4 and SNAP-23 [9]. This reaction is not constitutive. It is tightly regulated by accessory proteins, among which the Sec1/Munc18-like protein Munc18c is particularly important [61]. Munc18c binds syntaxin-4 and helps govern the transition between restrained and fusion-permissive states [61].

In vitro and cellular studies support an inhibitory role for Munc18c in the basal state, where association with syntaxin-4 can prevent premature or unproductive SNARE assembly [9,61]. However, genetic perturbation data do not support an oversimplified model in which less Munc18c always means more effective GLUT4 exocytosis [61]. Homozygous and heterozygous perturbations have produced context-dependent phenotypes, indicating that Munc18c also performs organizing or permissive functions within the exocytic machinery [61]. This mechanism therefore requires cautious interpretation.

The system appears further specialized in skeletal muscle [62,63]. In vivo overexpression studies indicate that Munc18c can selectively impair GLUT4 translocation to transverse tubules without equivalently suppressing delivery to the sarcolemma, consistent with regional differences in syntaxin-4 abundance and membrane specialization [64]. This observation shows that the plasma membrane is not a uniform destination even within a single muscle fiber [62,63].

At least two additional regulatory events support efficient fusion [9,61]. First, insulin-stimulated phosphorylation of Munc18c at tyrosine 521 weakens interactions that otherwise stabilize a restrained state, favoring a more fusion-competent conformation [61]. Second, DOC2b translocates to the plasma membrane, where it has been proposed to facilitate SNARE assembly and promote local membrane curvature [9]. Together, these findings identify Munc18c as a critical gatekeeper in a process that is increasingly recognized as a synaptotagmin-independent exocytic pathway. In adipocytes, the hormone-triggered mobilization of GLUT4 is positively regulated by Complexin (Cpx), which accelerates the evoked phase of exocytosis without altering basal fusion [65]. Mechanistically, complexin promotes lipid bilayer remodeling via its C-terminal membrane-binding peptide, functioning as an evolutionary precursor to Ca^2+^-dependent synaptic release and offering a novel target for modulating insulin-sensitive glucose disposal [65].

### 6.5. Pathology of Insulin Resistance

In insulin-resistant states, glucose transport failure stems from distributed trafficking defects rather than protein depletion [9,57]. Recent studies report extracellular matrix stiffness as a novel regulator of this process; in human and rat adult cardiomyocytes, increased stiffness (as seen in heart failure) induces intracellular aggregation of GLUT4 within the microtubule network [66], blunting insulin-mediated uptake [66]. This mechanotransduction-related impairment is associated with stiffness-induced detyrosination of alpha-tubulin and can be partially rescued by AMPK activation [66], thereby lowering effective insulin-stimulated surface delivery [9,57]. Ceramide accumulation, altered ER-Golgi trafficking, and mis-sorting of GLUT4 into dense, less responsive endosomal pools have all been proposed as contributory mechanisms in selected systems [9].

Additional work suggests that stoichiometric imbalance among fusion-regulatory proteins may further impair exocytosis [9]. Examples include increased inhibitory Munc18c or accumulation of nonproductive SNARE combinations such as VAMP5/SNAP29-containing assemblies [9]. These observations support a view of insulin resistance as a distributed trafficking disorder rather than a defect confined to proximal receptor signaling [9,57] because GLUT4 translocation depends on a network of coupled events, partial dysfunction can arise at several levels without requiring catastrophic failure of any single component [9]. The field therefore does not support a simple inference from total GLUT4 abundance to actual glucose uptake capacity, especially in common metabolic disease [9].

## 7. Extended GLUT Family

The extended SLC2 family includes transporters with broader substrate selectivity and less fully defined physiology than GLUT1 through GLUT4 [6,7]. Some members are reasonably well characterized, whereas others remain functionally underdefined [7]. A balanced review should therefore avoid treating all extended GLUTs as equivalent in maturity or confidence [6,7].

GLUT5 is the principal facilitative fructose transporter in the small intestine [10]. Its expression is regulated by developmental state, dietary composition, cyclic AMP signaling, glucocorticoids, and in some models by nuclear receptor pathways [10]. The importance of GLUT5 in apical intestinal fructose handling is well established, and it provides a clear example of how the SLC2 family diversified beyond glucose [10]. However, broader claims linking GLUT5 uniformly to metabolic syndrome or multiple cancers should be calibrated carefully, because the magnitude and functional meaning of altered expression are highly context dependent [67]. The functional repertoire of the extended family has also been applied in adoptive immunotherapy. In hostile tumor microenvironments characterized by glucose depletion, the engineering of CAR-T cells to express GLUT5 provides a competitive metabolic advantage. By enabling these cells to utilize fructose as an alternative glycolytic substrate, the GLUT5 axis restores metabolic fitness, enhances migratory velocity, and sustains anti-tumor durability in glucose-deprived milieus [68].

GLUT6 and GLUT8 illustrate the challenges of class III transporter biology [7]. Both display substantial intracellular localization rather than the classical constitutive plasma membrane profile of GLUT1 or GLUT3 [7]. GLUT6 has been localized to lysosome-related compartments and shown to respond to inflammatory signaling in macrophage-associated systems, raising the possibility that it participates in recycling of substrates generated by intracellular degradation pathways [7]. GLUT8 also depends strongly on intracellular targeting signals and is found in endosomal or trans-Golgi-associated compartments in several models [7]. These findings are mechanistically interesting, but the broader physiological and pathological relevance of either transporter remains incompletely defined [7].

Transporter-mediated metabolic reprogramming is also a decisive factor in macrophage polarization and trained immunity. High-throughput proteomics has identified GLUT1 as the primary driver of pro-inflammatory (M1) phenotypes in conditions ranging from allergic rhinitis to sepsis-associated acute kidney injury [48,69]. Conversely, the delivery of specific microRNAs (e.g., miR-574-5p) via mesenchymal stem cell-derived extracellular vesicles can suppress GLUT1 expression, thereby restraining glycolysis and priming macrophages toward a pro-resolving M2 phenotype [48]. Modulating hexose flux is not merely a strategy for glycemic control but a precision tool for recalibrating systemic and tissue-specific inflammatory responses [48,69,70].

GLUT7, GLUT9, and GLUT11 illustrate a spectrum from relatively mature to still developing functional definition [7]. GLUT9 is supported as a urate transporter rather than a simple glucose carrier, and structural work has clarified important aspects of urate recognition and inhibitor binding [32]. GLUT7 and GLUT11, by contrast, remain less well resolved physiologically [7]. Although both have been associated with transport of glucose or fructose in heterologous systems, their integrated tissue-level roles are still not defined with the same confidence as those of GLUT5 or GLUT9 [7,32].

GLUT10, GLUT12, HMIT/GLUT13, and GLUT14 remain relevant but variably characterized [6,7]. HMIT is better regarded as a proton-coupled myo-inositol transporter than as a canonical glucose transporter [6]. GLUT12 has attracted interest in insulin sensitivity, muscle metabolism, and cancer biology, but evidence remains insufficient for strong generalizations [7,16]. GLUT10 has been linked to arterial tortuosity syndrome and may participate in dehydroascorbate transport or redox-related intracellular processes [7]. GLUT10 contributes to intracellular ascorbic acid (AA) homeostasis through a noncanonical trafficking route [33]. Under oxidative stress, GLUT10 redistributes from the endoplasmic reticulum to the plasma membrane to enhance dehydroascorbic acid (DHA) uptake, ultimately targeting mitochondria to sustain antioxidant capacity and support mitochondrial adaptation, thus bridging endomembrane trafficking and redox regulation [33]. GLUT14 appears to be a relatively recent duplication related to GLUT3 with prominent testicular expression, but its broader physiological significance remains limited [7].

**Table 2:** Structural characteristics, substrate profiles, and physiological relevance of the SLC5 secondary-active cotransporter family and related transporters.

Transporter Isoform	Gene Symbol	Primary Substrates	Tissue Distribution	Structural and Functional Characteristics
SGLT1 [2]	SLC5A1	Glucose, galactose, water, urea	Small intestine, renal S3 segment	High-affinity cotransporter (2Na^+^); mediates water/urea transport via channel activity.
SGLT2 [38,71]	SLC5A2	Glucose, sodium	Renal S1 and S2 segments	Low-affinity, high-capacity cotransporter (Na^+^); reabsorbs 90% approximately of filtered renal glucose.
SGLT3 [8]	SLC5A4	Sodium ions (glucose-activated)	Neuromuscular junctions, enteric nervous system	Glucose-activated ion channel/sensor; does not perform net sugar transport.
SMIT1 [3]	SLC5A3	Myo-inositol, sodium	Brain, kidney, ubiquitous	Osmoregulatory transporter; encoded by a unique single-exon gene.
CHT [3]	SLC5A7	Choline, sodium, chloride	Cholinergic nerve terminals	Sodium/chloride-coupled choline cotransporter; rate-limiting step in acetylcholine synthesis.
SMCT1 [3]	SLC5A8	Monocarboxylates, sodium	Kidney, colon, thyroid	High-affinity transporter for lactate, pyruvate, and short-chain fatty acids.
SGLT5 [72,73]	SLC5A10	Fructose, mannose	Kidney	Required for fructose-mediated salt retention and proximal nephron oxidative stress
SWEET1 [1,3]	SLC50A1	Glucose (facilitated efflux)	Ubiquitous (low expression)	Single-member class in humans; mediates facilitated sugar efflux

## 8. SGLT1, SGLT2, and SGLT3

SGLT1 and SGLT2 are the principal sodium-dependent glucose cotransporters in mammalian physiology and the clearest examples of epithelial glucose transport by the SLC5 family [2,8]. SGLT1 mediates apical uptake of glucose and galactose in the small intestine and also contributes to renal glucose recovery in the distal part of the proximal tubule, whereas SGLT2 is the dominant transporter responsible for bulk glucose reabsorption in the early proximal tubule [2,10]. Their functional complementarity with basolateral GLUT2 provides a classic example of vectorial transport [2,10].

In the kidney, glucose reabsorption is spatially partitioned along the proximal tubule [2]. SGLT2, a low-affinity, high-capacity cotransporter, is localized mainly to the apical membrane of the S1 and S2 segments and accounts for most filtered glucose recovery under euglycemic conditions [2]. SGLT1, a higher-affinity transporter, recovers most of the remainder in the S3 segment [2]. This arrangement is physiologically efficient because it combines bulk early reclamation with a downstream safety net capable of scavenging residual luminal glucose at lower concentration [2].

In the intestine, the general framework is similarly well established [10]. SGLT1 mediates most apical uptake of glucose and galactose, while GLUT2 supports basolateral efflux [5,10]. Considerable attention has been given to the possibility of acute apical recruitment of GLUT2 in response to high luminal sugar loads [10]. Some studies have supported this model, whereas others have emphasized the continued dominance of SGLT1 even under high luminal glucose exposure [5,10]. The most defensible interpretation is that apical GLUT2 recruitment remains a debated and likely context-dependent phenomenon rather than a universally dominant mechanism [10].

Human genetics confirms the nonredundant importance of these transporters [2]. Pathogenic variants in SLC5A1 cause glucose-galactose malabsorption, a severe intestinal disorder of neonatal carbohydrate absorption, whereas pathogenic variants in SLC5A2 cause familial renal glucosuria, in which glucose appears in the urine despite otherwise preserved renal function [2]. These disorders provide direct evidence that intestinal SGLT1 and renal SGLT2 perform distinct essential epithelial functions in vivo [2]. The functional scope of the SLC5 family must now include SGLT5 as a primary mediator of diet-induced hypertension. Recent evidence demonstrates that high-fructose intake upregulates renal SGLT5 expression, which is required for fructose-mediated salt retention and the augmentation of Angiotensin II-stimulated proximal nephron oxidative stress [72,73]. SGLT5-mediated fructose reabsorption initiates a genetic program that drives salt-sensitive hypertension, establishing this transporter as a distinct therapeutic target in metabolic-related vascular disease [72,73].

**Figure 5:**
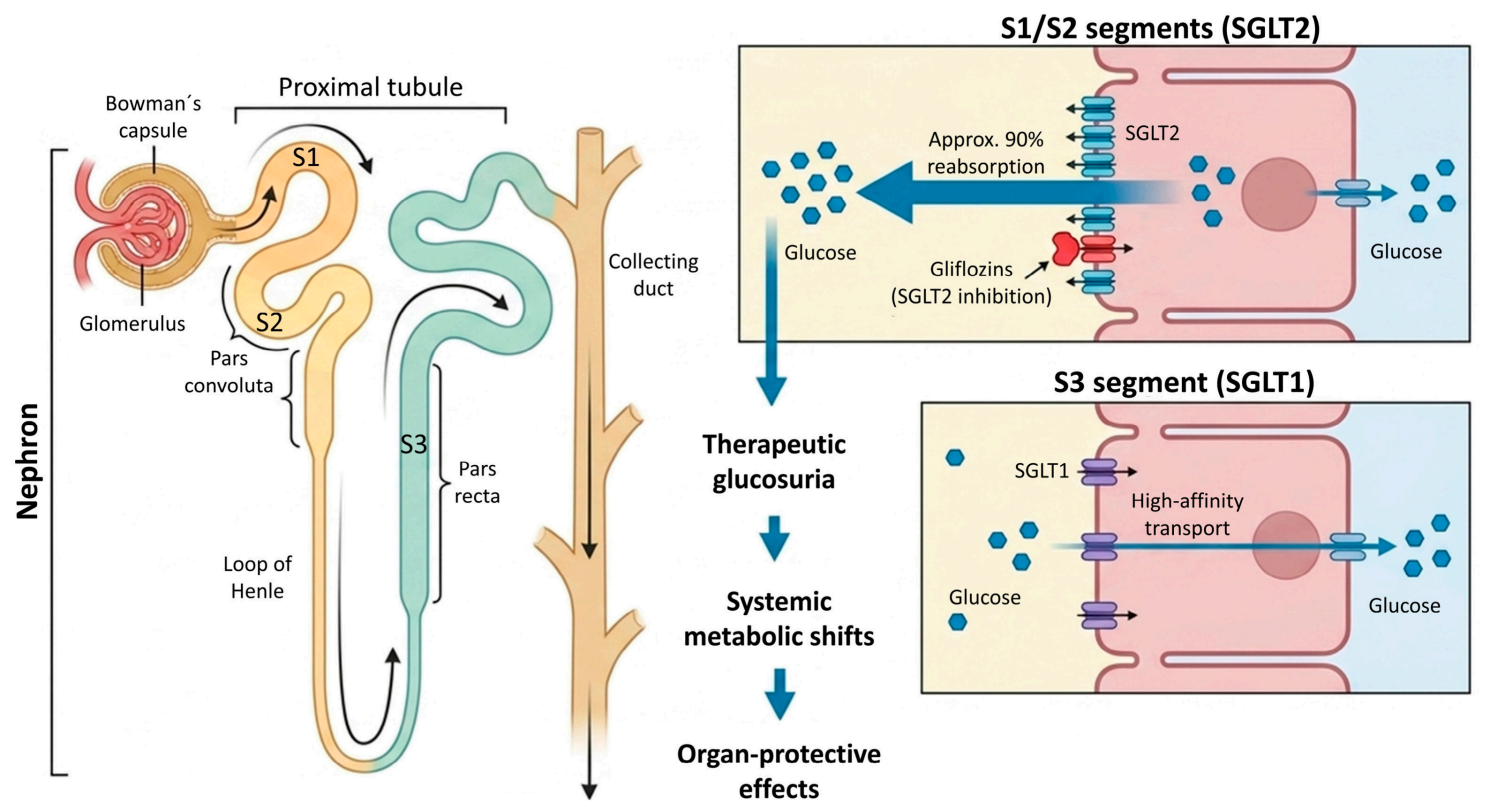
Segment-specific glucose reabsorption in the renal proximal tubule. Functional anatomy of the nephron showing the spatial partitioning of glucose recovery. SGLT2, located in the S1 and S2 segments, facilitates approximately 90% of filtered glucose reabsorption. The remaining glucose is recovered by the high-affinity SGLT1 transporter in the distal S3 segment. Pharmacological inhibition of SGLT2 by gliflozins promotes therapeutic glucosuria, triggering systemic metabolic shifts and downstream organ-protective effects. Abbreviations: GLUT, glucose transporter; SGLT, sodium-glucose cotransporter.

SGLT3 is a functional outlier [8]. Although structurally related to SGLT1, it does not appear to mediate substantial net glucose flux [8]. Instead, glucose binding triggers sodium-dependent membrane depolarization, supporting the view that SGLT3 functions primarily as a glucose sensor or sensor-like protein [8]. This divergence shows how a transporter family can evolve sensing functions while retaining structural features associated with solute recognition [8] (Figure 5).

Therapeutically, inhibition of SGLT2 has become a major advance in diabetology, nephrology, and cardiovascular medicine [2,74]. The original rationale was induction of glucosuria to lower plasma glucose independently of insulin [74]. Clinical benefits of SGLT2 inhibitors extend beyond glucosuria through sophisticated immunometabolic interactions. While inducing glycosuria, SGLT2 inhibition maintains the kidney’s antibacterial response by decreasing complement C1q expression in the proximal tubules, which modulates the MERTK pathway in myeloid cells and preserves monocyte-mediated neutrophil chemotaxis despite glycosuria [75,76]. This immune-metabolic homeostasis may explain why SGLT2i do not increase the risk of severe pyelonephritis in clinical cohorts [76,77,78]. This mechanism has been proposed to explain the sustained renal defense observed in clinical practice despite increased urinary glucose availability [76]. Notably, these agents act as allosteric activators of the mitochondrial enzyme HMGCS2, directly stimulating liver ketogenesis independently of the insulin-to-glucagon ratio [18,54,75,77]. This metabolic shift provides β-hydroxybutyrate, which functions as both an efficient fuel and an anti-inflammatory signaling molecule [75,77]. Furthermore, empagliflozin has been shown to directly bind and downregulate Aquaporin-1 (AQP1) in cardiomyocytes, ameliorating myocardial edema and restoring ion transporter balance (NHE1, NCX1), which improves mitochondrial function in prediabetic HFpEF models [79]. SGLT1 inhibition has also attracted interest because of its influence on intestinal glucose absorption, postprandial physiology, and incretin-related signaling [80].

## 9. Regulation of Glucose Transporters

Regulation of glucose transporters occurs at multiple levels, including gene transcription, mRNA stability, post-translational modification, subcellular localization, vesicular sorting, membrane insertion, endocytosis, and membrane microenvironment [9,81]. Acute regulation and chronic regulation should be distinguished because they often involve different biological questions. Acute regulation typically concerns how transporters are mobilized or activated over minutes to hours, whereas chronic regulation concerns how tissue expression patterns adapt to development, diet, hormones, hypoxia, or disease.

At the transcriptional level, transporters respond to tissue context, nutrient status, and cellular stress [10,16]. GLUT1 and GLUT3 can be upregulated in proliferative or hypoxic settings, consistent with their role in supporting increased glycolytic demand [16]. Intestinal GLUT5 and SGLT1 respond to dietary carbohydrate exposure and developmental stage [10]. Some class III GLUTs appear responsive to inflammatory or stress-related signaling [7]. Although these observations are informative, they should not be generalized indiscriminately across tissues, because similar upstream stimuli can lead to quite different transporter responses depending on cell identity and chromatin context [7,16].

At the trafficking level, GLUT4 remains the paradigm [9]. Insulin does not simply increase catalytic activity of preexisting surface transporters; it reorganizes intracellular membrane traffic to shift GLUT4 from stored intracellular pools to the plasma membrane [9,29]. This process depends on vesicle sequestration, TUG-mediated anchoring, Akt-AS160-Rab signaling, and SNARE-mediated fusion, making it a canonical example of transporter regulation through membrane traffic rather than transcriptional induction alone [9,59].

Membrane environment provides another layer of regulation [81]. Reconstitution studies show that transporter conformation and activity can depend on local lipid composition, especially phospholipid headgroups and bilayer properties [81]. These observations indicate that transporters do not operate in a vacuum. However, the direct extrapolation of reconstituted lipid effects to intact tissues remains limited by the complexity of native membranes, which contain dynamic microdomains, interacting proteins, and cytoskeletal constraints that are difficult to reproduce in simplified systems [81].

Taken together, these layers of control show why transporter abundance alone is often an inadequate predictor of physiological glucose flux [9,81]. The effective behavior of a transporter depends on where it is, what state it occupies, what proteins it interacts with, and how the surrounding membrane and signaling context shape its access to substrate [9,81]. This is particularly relevant in insulin-responsive tissues and increasingly important when interpreting disease-related expression data in cancer or inflammatory biology [9,16].

### Dietary Factors and Polyphenols

Beyond hormonal and metabolic cues, dietary components exert a significant regulatory role on glucose transporters. In particular, polyphenols act as potent modulators via direct inhibition, downregulation of expression, or prevention of plasma membrane translocation. Molecules such as quercetin and epigallocatechin gallate interfere with GLUT-mediated uptake and SGLT-driven cotransport, offering a nutritional strategy to manage glycemic excursions [3,82]. Over direct inhibition, dietary polyphenols act as metabolic rheostats through complex signaling networks. For instance, rutin and extracts from *Smilax china* L. and bergamot juice have been shown to upregulate GLUT4 expression in skeletal muscle and adipose tissue via the IRS/AKT-AMPK signaling pathway, effectively counteracting high-fat diet-induced insulin resistance [83,84,85]. In the liver, bioactive compounds like those found in lychee honey and carob fruit extract modulate the ChREBP/GLUT4 and insulin receptor β/PI3K/Akt axes, enhancing hepatic glucose uptake and regenerating pancreatic β-cells [86,87]. Furthermore, chlorogenic acid (CGA) exerts a tissue-specific effect; while it limits sugar surges, it also increases intestinal SGLT1 and GLUT2 expression to reinforce mucosal barrier integrity and attenuate oxidative stress [88,89]. Notably, resveratrol can synergize with SGLT2 inhibitors by attenuating dapagliflozin-induced renal gluconeogenesis, a common compensatory mechanism, through the activation of the PI3K/Akt pathway and suppression of FoxO1, thereby optimizing the overall glucose-lowering efficacy [90].

## 10. Disease Relevance and Therapeutic Implications

Monogenic transporter disorders provide some of the clearest causal links between transporter dysfunction and human disease [2,24]. GLUT1 deficiency syndrome, Fanconi-Bickel syndrome, glucose-galactose malabsorption, and familial renal glucosuria each identify a specific transporter as physiologically indispensable in a defined tissue context [2,24]. These disorders also illustrate that the phenotype of transporter dysfunction depends strongly on normal tissue distribution. A defect in a blood–brain barrier transporter yields a neurological syndrome; a defect in an epithelial cotransporter yields malabsorption or glucosuria; a defect in a broadly distributed metabolic transporter yields multisystem consequences [2,24,26].

In common acquired disease, however, transporter pathology is often more subtle [9,57]. Rather than complete loss of function, one often sees altered expression, defective trafficking, mislocalization, dysregulated substrate preference, or remodeling of interacting networks [9,16]. This is especially true in insulin resistance, where impaired GLUT4 vesicle sorting, anchoring, trafficking, and fusion can lower glucose uptake without eliminating the transporter itself [9].

Cancer-related transporter biology is also important but requires caution [16,67]. Upregulation of GLUT1 and GLUT3 is common in highly glycolytic tumors and may support increased glucose demand, but the biological meaning of increased expression is not uniform across malignancies [16]. For some transporters, especially the less well-characterized extended GLUTs, disease associations are often ahead of mechanistic resolution [7,67]. This does not make such associations uninteresting, but it does argue against overstating therapeutic implications before functional necessity is demonstrated in the relevant tumor context [16].

### 10.1. Transporter Defects as Disease Mechanisms

Transporter-related disease mechanisms can be grouped broadly into three categories: loss-of-function disorders, dysregulated trafficking, and pathological overexpression or repurposing in acquired disease [9,16,24]. Loss-of-function disorders are most clearly exemplified by inherited syndromes such as GLUT1 deficiency and Fanconi-Bickel syndrome, in which the causal relationship between transporter dysfunction and phenotype is direct and compelling [24,26]. Dysregulated trafficking is best illustrated by GLUT4 in insulin resistance, where defective membrane traffic rather than absent protein is the dominant issue [9]. A third category involves pathological metabolic ‘addictions’ in which transporter overexpression creates collateral vulnerabilities. An example is the disulfidptosis pathway in SLC7A11-high tumors. These cells rely heavily on GLUT1-mediated glucose uptake to generate NADPH for reducing intracellular cystine. Recent studies describe a form of regulated cell death termed disulfidptosis, a metabolism-dependent cell death modality unique to SLC7A11-high tumors. Under glucose limitation or GLUT1 inhibition, the depletion of NADPH prevents the reduction of intracellular cystine, leading to toxic disulfide bond accumulation and the irreversible collapse of the actin cytoskeleton [91,92]. This observation suggests a potential therapeutic strategy: while SLC7A11 supports glutathione synthesis to resist ferroptosis, it simultaneously creates an ‘Achilles’ heel’ of glucose-derived NADPH addiction that can be exploited for precision oncology [91,92,93]. Additionally, GLUT1 and MCT1 dependencies have been implicated in promoting resistance to neoadjuvant chemoradiotherapy in colorectal cancer, where metabolic reprogramming facilitates rapid DNA repair and survival [16,94]. In pancreatic cancer, the intracellular enzyme paraoxonase 2 (PON2) has been identified as a novel regulator of GLUT1; PON2 promotes tumor growth and metastasis by stimulating GLUT1-mediated glucose transport and glycolytic flux [95].

This classification distinguishes why “transporter disease” is not a single mechanistic concept [9,24]. The same family can yield Mendelian syndromes, complex post-translational disorders, or secondary expression signatures depending on tissue context and disease type [2,16]. Mechanistic interpretation should therefore remain sensitive to the level at which dysfunction arises [9].

**Figure 6:**
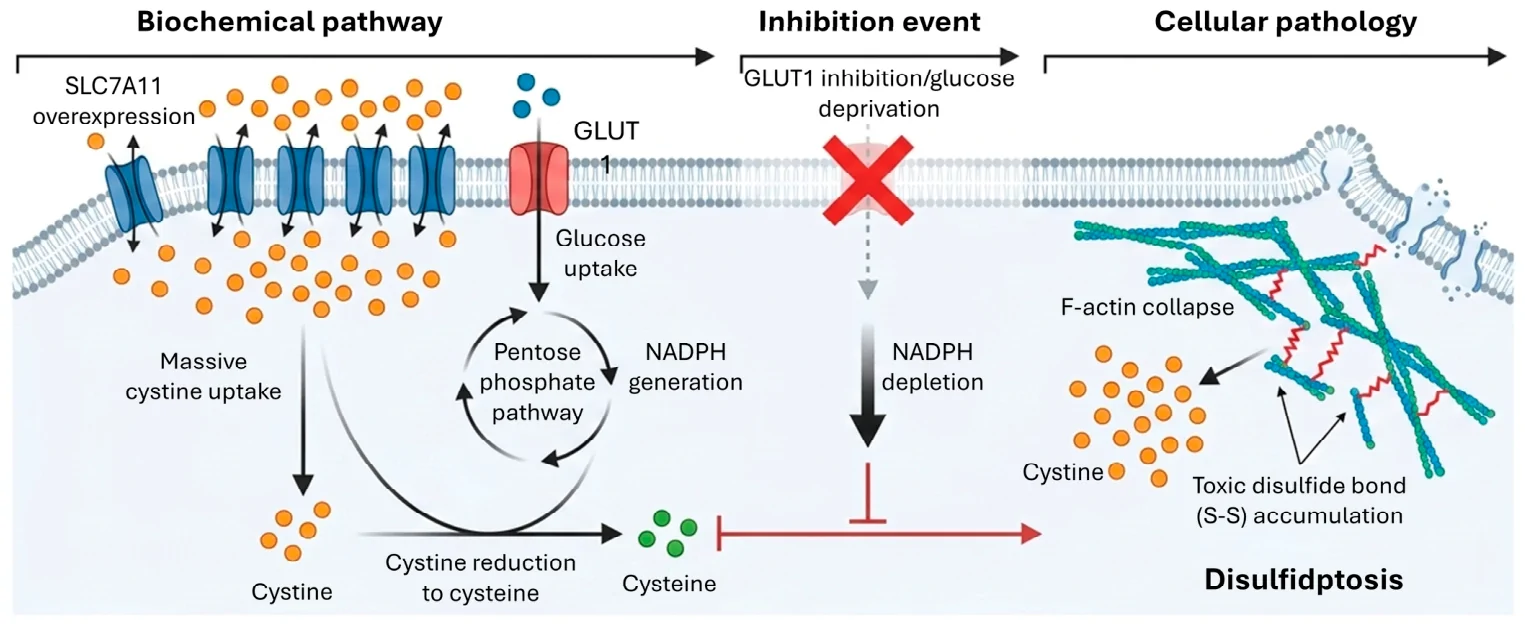
Mechanism of disulfidptosis in SLC7A11-overexpressing cancer cells. High levels of the cystine/glutamate transporter SLC7A11 drive massive cystine uptake, creating a critical dependency on GLUT1-mediated glucose uptake to generate NADPH via the pentose phosphate pathway. Under glucose deprivation or GLUT1 inhibition, NADPH depletion prevents cystine reduction, leading to toxic disulfide bond accumulation among actin cytoskeletal proteins, F-actin collapse, and a unique form of regulated cell death termed disulfidptosis. Abbreviations: GLUT1, glucose transporter 1; NADPH, nicotinamide adenine dinucleotide phosphate; SLC7A11, solute carrier family 7 member 11 (xCT).

Beyond aerobic glycolysis, a novel form of regulated cell death termed disulfidptosis has emerged as a metabolic vulnerability in SLC7A11-high tumors [92,96]. These cells become ‘addicted’ to glucose-derived NADPH to reduce toxic intracellular cystine; consequently, targeted inhibition of GLUT1 (e.g., with BAY-876) selectively triggers actin cytoskeleton collapse and death in these treatment-resistant solid tumors. Critically, this metabolic vulnerability extends to the tumor microenvironment, where intratumoral CD8+ T cells also undergo SLC7A11-dependent disulfidptosis under glucose limitation, contributing to T-cell exhaustion and immune evasion [92]. Thus, modulating hexose flux via GLUT1 represents a dual-action strategy to both eliminate malignant cells and potentially rejuvenate anti-tumor immunity by disrupting this metabolic checkpoint [92,93,96] (Figure 6).

### 10.2. Dual SGLT1/2 Inhibition with Sotagliflozin

Sotagliflozin is a dual inhibitor of SGLT1 and SGLT2 and remains the clearest clinical example of combined intestinal and renal sodium-glucose cotransporter inhibition [80]. It is substantially more potent against SGLT2 than SGLT1, allowing effective renal blockade at therapeutic doses while still exerting functionally relevant inhibition of intestinal SGLT1 [80].

This dual mechanism introduces several physiological consequences that differ in part from those of selective SGLT2 inhibitors [80]. By inhibiting intestinal SGLT1, sotagliflozin delays and reduces proximal glucose absorption, attenuates postprandial glycemic excursions, and may lower glycemic variability [80]. Increased distal glucose delivery can in turn augment incretin-related signaling, including GLP-1 and peptide YY responses [80]. These pharmacological differences should be interpreted, but they are best framed as pharmacological distinctions rather than as proof of universal clinical superiority over selective SGLT2 inhibition [80] (Table 3).

Sotagliflozin has also been studied as adjunctive therapy in type 1 diabetes [80]. In that setting, potential improvements in glycemic control must be weighed against increased risk of diabetic ketoacidosis, illustrating a broader principle in transporter-targeted therapy: mechanistic elegance does not eliminate the need for disease-specific safety calibration [80].

### 10.3. Cardiac Energetics and Cellular Stress Pathways

The cardiovascular benefits of SGLT inhibition cannot be explained solely by renal glucose lowering, and this has motivated a large mechanistic literature [74,78,97]. Proposed explanations include osmotic and natriuretic effects, reduced preload and afterload, improved renal-hemodynamic coupling, altered substrate selection, modulation of inflammation, attenuation of oxidative stress, and direct or indirect effects on cardiac ion handling [74,98].

One influential hypothesis is the so-called thrifty fuel model, according to which SGLT inhibition alters systemic fuel availability and modestly favors ketone body use by the myocardium [74]. In principle, ketones may represent an efficient auxiliary fuel under selected conditions. The idea is attractive, but the magnitude and sufficiency of this mechanism remain debated, and it should not be treated as a complete explanation for the cardiorenal benefits of the drug class [74].

Experimental work has also suggested that some SGLT2 inhibitors can modulate the cardiac Na^+^/H^+^ exchanger NHE1, thereby lowering intracellular Na+ and secondarily affecting Ca^2+^ handling, mitochondrial stress, and reactive oxygen species generation [98]. Related literature has linked these effects to reduced autosis, improved remodeling, and enhanced post-injury cardiac performance in experimental systems [98]. These findings are mechanistically plausible, but a class-wide explanation for clinical outcomes is not uniformly established [98]. The clearest conclusion is that heart-failure-related benefits are clinically well supported, whereas the relative contribution of specific intracellular mechanisms is still being resolved [78,97].

### 10.4. Clinical Trial Evidence

The clinical efficacy of sotagliflozin in cardiovascular and cardiorenal disease is supported primarily by the SOLOIST-WHF and SCORED trials [78,97]. Together, these studies showed that dual SGLT1/2 inhibition can reduce clinically relevant heart-failure-related outcomes in high-risk populations with diabetes.

In SOLOIST-WHF, sotagliflozin was started before or shortly after discharge in patients with recent worsening heart failure and reduced the total burden of cardiovascular death, heart-failure hospitalization, and urgent heart-failure visits compared with placebo, with a hazard ratio of 0.67 and a 95% confidence interval of 0.52 to 0.85 [97]. This finding is notable because it suggests benefit in a recently decompensated population, a high-risk clinical setting [97].

In SCORED, sotagliflozin reduced the revised primary composite of cardiovascular death and heart failure events in patients with type 2 diabetes and chronic kidney disease [78]. Beyond clinical efficacy, the safety profile of SGLT inhibition regarding urinary tract infections has been elucidated; SGLT2i therapy maintains the renal antibacterial response by decreasing complement C1q expression and modulating the MERTK pathway in myeloid cells, preserving monocyte-mediated neutrophil chemotaxis despite increased glycosuria [76]. Consequently, these agents function as fundamental disease-modifying therapies that balance metabolic control with immune homeostasis [76,78].

Recent evidence indicates that SGLT2i-mediated metabolic benefits may involve direct activation of hepatic ketogenesis. Gliflozins act as allosteric activators of the mitochondrial enzyme HMGCS2 (3-hydroxy-3-methylglutaryl-CoA synthase 2), the rate-limiting step in ketone body synthesis [77]. This direct enzymatic modulation, independent of the insulin-to-glucagon ratio, ensures a sustained supply of β-hydroxybutyrate, which serves as both an efficient fuel for the failing heart and an anti-inflammatory signaling molecule [75,77].

### 10.5. Cardio-Oncology

Interest in SGLT inhibition has increasingly extended into cardio-oncology, particularly for prevention or attenuation of treatment-related cardiac dysfunction [17]. This interest is biologically plausible because many cardiotoxic anticancer therapies induce oxidative stress, inflammation, fibrosis, endothelial injury, and progressive ventricular dysfunction [17].

Recent evidence identifies SGLT2 inhibitors as potent mitigators of chemotherapy-induced cardiotoxicity by preserving transverse tubule integrity and optimizing Ca2+ handling in mice and rat-derived H9C2 cells [99,100]. The vasculoprotective scope of these agents now includes pulmonary arterial hypertension, where empagliflozin directly binds to the tyrosine kinase effector domain of PDGFR-beta, inhibiting its phosphorylation and attenuating pathological vascular remodeling [101]. This suggests that the cardio-oncological benefits of SGLT2i are not limited to cardiomyocyte survival but extend to direct modulation of vascular growth factor signaling [99,100,101]. These agents attenuate doxorubicin-induced dyssynchronous sarcoplasmic reticulum calcium release and promote cardiomyocyte survival through the activation of the AMPK/mTOR and SIRT1/PGC-1α signaling pathways, thereby reducing mitochondrial dysfunction and inflammatory cytokine production [17,100,102].

For that reason, translational enthusiasm should be proportional to evidence strength. Emerging cardio-oncology findings are promising, but they should not be translated into strong drug-specific claims for sotagliflozin unless supported directly. The same caution applies to related observations such as reductions in epicardial adipose tissue, which should be attributed only to the specific agents and studies from which they derive [103,104].

In clinical oncology, the competition for nutrients between malignant cells and infiltrating lymphocytes has identified specific transporter dependencies as critical therapeutic targets. While tumors utilize GLUT1-mediated glycolysis to drive the Warburg effect, effector CD8+ T cells selectively require GLUT10 to sustain their activation and anti-tumor efficacy [105]. Crucially, the TME-accumulated lactic acid functions as a metabolic checkpoint by directly binding to an intracellular motif of GLUT10, thereby inhibiting T-cell glucose uptake and promoting immune evasion [105]. Complementing this, synthetic biology approaches now leverage substrate flexibility; engineering CAR-T cells to express GLUT5 allows these cells to bypass glucose deprivation by utilizing fructose as an alternative fuel, thereby restoring metabolic fitness and enhancing ‘hit-and-run’ serial killing in hostile, glucose-depleted environments [68].

**Table 3:** Functional and clinical distinctions between sotagliflozin and selective SGLT2 inhibitors.

Feature/Parameter	Sotagliflozin	Selective SGLT2 Inhibitors
Primary Mechanisms	Apical SGLT2 inhibition in S1/S2 renal segments, and SGLT1 inhibition in S3 renal segment and proximal jejunum [80].	Apical SGLT2 inhibition in S1/S2 renal segments, sparing SGLT1 under clinical doses [77].
Selectivity Ratio	Approximately 20-fold selectivity for SGLT2 over SGLT1.	>selectivity for SGLT2 over SGLT1.
Renal Target Segment	S1, S2, and S3 segments of the proximal tubule.	S1 and S2 segments of the proximal tubule.
Intestinal Target	Local inhibition of enterocyte apical SGLT1.	None.
Endocrine Effects	Stimulates L-cell secretion of GLP-1 and PYY.	No direct impact on intestinal L-cell incretin release.
Clinical Trial Evidence	SOLOIST-WHF: HR 0.67 for CV death, HF hospitalizations, and urgent HF visits in patients post-worsening HF.52 SCORED: HR 0.74 in patients with T2D and CKD [78,97].	20% reduction in worsening HF or CV death within 90 days; rapid symptom relief by day 5.44 Post-MI [106] 14% reduction in all-cause mortality [107].
Ischemic Reductions	Significant reductions in ischemic outcomes: Myocardial Infarction HR 0.68; Stroke HR 0.66.	Consistent reduction in HF-related outcomes but generally lacks statistically significant reductions in ischemic MI or stroke events.
Cardio-Oncology Efficacy	Influences metabolic markers; may reduce epicardial adipose tissue.	53% reduction in all-cause mortality (RR 0.47) and 52% reduction in HF composite (RR 0.48) in cardiotoxic chemotherapy [108].
Adverse Event Profile	Increased incidence of mild diarrhea and volume depletion.	Lower incidence of GI adverse events; rare cases of euglycemic ketoacidosis [77].

## 11. Experimental Approaches

Glucose transporter biology is studied using a combination of radiotracer uptake assays, heterologous expression systems, oocyte platforms, membrane reconstitution, live-cell imaging, super-resolution microscopy, biochemical fractionation, mutagenesis, electrophysiology, and structural methods including crystallography and cryo-EM [1,4,81]. Each approach answers different questions and carries different limitations.

Heterologous systems are especially valuable for defining substrate specificity, inhibitor sensitivity, and kinetic parameters under controlled conditions [3,4]. Xenopus oocytes and transfected mammalian cells have been used extensively for SGLT and GLUT studies because they permit direct comparison of wild-type and mutant transporters in a tractable setting [3]. However, these systems may not reproduce native trafficking circuits, membrane lipid composition, or tissue-specific protein partnerships [81].

Biochemical and imaging methods have been particularly important in the GLUT4 field [9,57]. Subcellular fractionation, immunoisolation, total internal reflection fluorescence microscopy, and more recent dynamic imaging platforms have each revealed different aspects of vesicle identity, mobility, docking, and fusion [9,57]. Structural biology, meanwhile, has transformed interpretation of both GLUT and SGLT transporters by clarifying conformational states, substrate-binding environments, ion coupling logic, and inhibitor interactions [1,38].

A central interpretive issue across all these methods is that no single platform captures mechanism, compartmental organization, tissue context, and human relevance simultaneously [1,9]. Conclusions are therefore strongest when convergent evidence from structural, biochemical, cell-biological, physiological, and clinical studies points in the same direction [1,78].

## 12. Outstanding Questions and Future Perspectives

Despite major progress, several transporters remain incompletely characterized, particularly among the extended GLUT family and selected members of the broader SLC5 family [6,7]. For some proteins, substrate specificity remains incompletely resolved; for others, intracellular localization is clearer than physiological function. Disease associations also often outpace mechanistic validation [16,67].

In intestinal physiology, the extent and functional significance of acute apical GLUT2 recruitment remain unsettled [5,10]. In pancreatic biology, the challenge is not simply cataloguing transporter expression, but understanding how species differences alter interpretation of β-cell glucose sensing [22]. In insulin-responsive tissues, the central unresolved question is no longer whether GLUT4 trafficking is regulated, but exactly how specialized storage vesicles, endosomal intermediates, Rab circuits, cytoskeletal transport, and plasma membrane fusion are integrated into a coherent dynamic system across different cell types and physiological states [9,57].

In translational medicine, the field still needs a clearer separation between well-established therapeutic outcomes and more speculative mechanistic narratives [78,97,98]. The success of SGLT inhibition in cardiorenal disease is now clinically secure, but several proposed downstream mechanisms remain only partly resolved [78,97]. Likewise, expression-based disease associations for less well-characterized transporters should not be allowed to substitute for direct evidence of functional necessity [16,67].

Future work will likely combine high-resolution structure, quantitative live-cell trafficking, tissue-specific physiology, human genetics, and clinical translation. [1,38]. Studies may focus less on discovering entirely new transporter families and more on understanding how known transporters are embedded in dynamic cellular and organ-level systems [1,9]. Therapeutic expansion into neurodegeneration is supported by recent evidence showing that SGLT2 inhibition is associated with a significant reduction in incident all-cause dementia (OR 0.61) and Alzheimer’s disease risk [109,110]. These effects may be mediated by improvements in regional brain metabolism, blood–brain barrier stability, and attenuation of neuroinflammation and amyloid-beta aggregation [111,112]. In the realm of cardiorenal protection, SGLT2 inhibitors have demonstrated a consistent erythropoietic stimulatory effect, increasing hemoglobin and hematocrit across both diabetic and non-diabetic populations [113,114]. This response does not appear to be due solely to hemoconcentration and may involve a coordinated iron-metabolic axis. SGLT2i therapy enhances the erythropoietic response to oral iron supplementation in patients with heart failure and CKD, potentially by modulating hepcidin-mediated transport barriers and alleviating pro-inflammatory suppression of the bone marrow [77,114,115]. Furthermore, the clinical applications for SGLT2 inhibitors is expanding into rare genetic and autoimmune conditions. In Alport syndrome, SGLT2 inhibition combined with ACE inhibitors has shown promise in preventing irreversible kidney damage by reducing proteinuria and preserving glomerular basement membrane integrity [116]. Similarly, in patients with lupus nephritis and residual proteinuria, SGLT2i therapy significantly improves eGFR slopes and reduces inflammatory markers, independent of the initial proteinuric response [115].

Additionally, GLP-1 receptor activation on astrocytes promotes astrocyte-neuron lactate and lipid coupling, sustaining aerobic glycolysis, which may contribute to neuroprotection for Alzheimer’s progression [117]. Future investigation is needed to understand the role of this neuroimmune coupling in acute and chronic brain dysfunction. Characterizing how systemic inflammation or metabolic stress, as seen in sepsis-associated encephalopathy (SAE), disrupts the microglial-CYR61 axis may reveal why glucose hypometabolism often precedes clinical decline [18,53]. Future strategies targeting this glial-vascular interface, rather than solely increasing systemic glycemia, may offer novel therapeutic avenues for neurodegenerative trajectories where activity-dependent fueling is compromised [18,53,112].

## 13. Conclusions

Mammalian glucose transporter research has progressed from initial membrane physiology to therapeutic application [1,38]. Structural, biochemical, and cell-biological studies have clarified major differences between facilitative uniporters and sodium-coupled cotransporters and have helped define how transporter localization, conformational cycling, intracellular sorting, and membrane environment shape tissue-specific glucose handling [4,9,81].

At the cellular level, work on transporter targeting and regulated traffic, particularly in the GLUT4 system, has shown how compartmentalization contributes to metabolic homeostasis and how disruption of these pathways may participate in insulin resistance [9,57]. Comparable advances in SGLT biology have clarified the segment-specific organization of renal glucose reabsorption, the structural basis of transporter inhibition, and the mechanistic diversity within the SLC5 family [2,38].

These advances are most clearly reflected in the therapeutic success of SGLT inhibition [78,97]. Clinical benefit in heart failure and chronic kidney disease is established, whereas the mechanisms linking transporter inhibition to broader cardiometabolic outcomes remain partly resolved and are likely multifactorial. [74,78,97]. Dual SGLT1/2 inhibition with sotagliflozin extends this translational framework by combining renal and intestinal actions, with demonstrated relevance particularly in heart-failure-related settings [78,80,97]. Furthermore, SGLT1 inhibition is emerging as a critical strategy in metabolic disorders and obesity. By delaying intestinal glucose absorption and promoting incretin release, SGLT1 targeting mitigates postprandial hyperglycemia and systemic inflammation. Recent evidence suggests that SGLT1 also plays a role in the intestinal inflammatory response, where its modulation can alleviate mucosal injury and preserve barrier integrity in models of enteritis [76,80].

The field supports two main conclusions. First, the core biology of major transporters such as GLUT1, GLUT2, GLUT3, GLUT4, SGLT1, and SGLT2 is now sufficiently mature to support deep integration of structure, physiology, and clinical relevance. Second, many important questions remain open, especially regarding intracellularly localized GLUTs, context-dependent transporter regulation, and the mechanistic interpretation of emerging therapeutic effects. Future work will be strongest when it preserves this balance between mechanistic ambition and evidentiary restraint.

## Data Availability

No new data were created or analyzed in this study. Data sharing is not applicable to this article.

## Abbreviations

The following abbreviations are used in this manuscript:

AA	Ascorbic acid
ACE	Angiotensin-converting enzyme
Akt	Protein kinase B
AMPK	AMP-activated protein kinase
AQP1	Aquaporin-1
AS160	Akt substrate of 160 kDa (also TBC1D4)
CAR-T	Chimeric antigen receptor T cell
CKD	Chronic kidney disease
CKM	Cardiovascular-kidney-metabolic syndrome
cryo-EM	Cryo-electron microscopy
CYR61	Cysteine-rich angiogenic inducer 61
DHA	Dehydroascorbic acid
DOC2b	Double C2-like domain beta
eGFR	Estimated glomerular filtration rate
G6P	Glucose-6-phosphate
G6PT	Glucose-6-phosphate transporter (SLC37A4)
GAP	GTPase-activating protein
GLP-1	Glucagon-like peptide-1
GLUT	Glucose transporter (SLC2 family)
GSV	GLUT4 storage vesicle
HF	Heart failure
HFpEF	Heart failure with preserved ejection fraction
HMIT	H^+^-myo-inositol transporter (also GLUT13)
HMGCS2	3-hydroxy-3-methylglutaryl-CoA synthase 2
IRAP	Insulin-regulated aminopeptidase
K8	Keratin 8
MAP17	Membrane-associated protein 17
MCT1	Monocarboxylate transporter 1
MFS	Major facilitator superfamily
mTOR	Mammalian target of rapamycin
Munc18c	Mammalian uncoordinated-18 homolog c
NCX1	Sodium-calcium exchanger 1
NHE1	Sodium-hydrogen exchanger 1
NLRP3	NLR family pyrin domain containing 3
PGC-1α	Peroxisome proliferator-activated receptor gamma coactivator 1-alpha
Pi	Inorganic phosphate
PIST	Golgi-associated PDZ-interacting protein
PRRSV	Porcine reproductive and respiratory syndrome virus
PYY	Peptide YY
Rab	Ras-related protein in brain
RIG-I	Retinoic acid-inducible gene I
ROS	Reactive oxygen species
SAE	Sepsis-associated encephalopathy
SCORED	Sotagliflozin in Patients with Diabetes and Chronic Kidney Disease
SGLT	Sodium-glucose cotransporter (SLC5 family)
SIRT1	Sirtuin 1
SLC2	Solute carrier family 2 (GLUT)
SLC5	Solute carrier family 5 (SGLT)
SLC37A4	Solute carrier family 37 member 4 (G6PT)
SLC50A1	Solute carrier family 50 member 1 (SWEET1)
SNAP-23	Synaptosomal-associated protein 23
SNARE	Soluble N-ethylmaleimide-sensitive factor attachment protein receptor
SOLOIST-WHF	Effect of Sotagliflozin on Cardiovascular Events in Patients With Type 2 Diabetes Post Worsening Heart Failure (trial)
SWEET	Sugars will eventually be exported transporter (SLC50A1)
T2D	Type 2 diabetes
TC10α	Rho-family GTPase
TD	Transport domain
TLR3	Toll-like receptor 3
TUG	Tether containing UBX domain for GLUT4
TXNIP	Thioredoxin-interacting protein
USP25m	Ubiquitin-specific protease 25m
VAMP2	Vesicle-associated membrane protein 2
